# Beyond the patient: how providers perceive and experience non-medical barriers in gastrointestinal cancer care

**DOI:** 10.1007/s00520-026-10897-3

**Published:** 2026-06-18

**Authors:** Trisha Lal, Daria Moody, Rishi Chiratanagandla, Gina Meehan, Christine Horvat Davey, Samudragupta Bora, Kurt Stange, Richard S. Hoehn

**Affiliations:** 1 Division of Surgical Oncology, University Hospitals, Cleveland Medical Center, 11100 Euclid Ave, Cleveland, OH 44106 USA; 2 School of Medicine, Case Western Reserve University, Cleveland, OH 44106 USA; 3 Case Comprehensive Cancer Center, Cleveland, OH 44106 USA; 4 Frances Payne Bolton School of Nursing, Case Western Reserve University, Cleveland, OH 44106 USA; 5 Health Services Research Center, University Hospitals Research & Education Institute, Cleveland, OH 44106 USA; 6 Department of Pediatrics, University Hospitals Rainbow Babies & Children’s Hospital, Case Western Reserve University School of Medicine, Cleveland, OH 44106 USA; 7 Center for Community Health Integration and Departments of Family Medicine and Community Health, Population and Quantitative Health Sciences, and Sociology, Case Western Reserve University, Cleveland, OH 44106 USA

**Keywords:** Non-medical barriers, Gastrointestinal cancer, Care coordination, Qualitative study

## Abstract

**Purpose:**

Gastrointestinal (GI) cancer care requires coordination across specialties and is sensitive to non-medical barriers like insurance, transportation, and health literacy. Prior survey work at our institution identified these barriers as most prominent during initial treatment planning and as consuming substantial provider time. This qualitative study represents the second phase of a larger effort to inform the development of a transdisciplinary care model designed to address non-medical determinants of care.

**Methods:**

We conducted semi-structured interviews with purposively sampled multidisciplinary providers involved in GI cancer care at a large academic health system in the USA. Interviews explored patient- and system-level barriers and opportunities to improve care coordination. Transcripts underwent inductive thematic analysis with double coding. Pearson correlation coefficients assessed covariation among themes and informed the construction of a directed acyclic graph illustrating hypothesized causal relationships among barrier domains.

**Results:**

We interviewed 30 providers, including physicians (*n* = 7), advanced practice providers (*n* = 2), nurses (*n* = 5), dietitians (*n* = 7), social workers (*n* = 6), and a care coordinator (*n* = 1). Seven interconnected themes emerged: health system, financial, access and logistics, provider capacity, patient knowledge and engagement, social and emotional, and communication and coordination. Structural and financial barriers operated as upstream drivers influencing workflow and access, while logistical and social barriers translated these pressures into downstream inefficiencies and provider strain. Providers consistently supported a transdisciplinary care model to centralize navigation and support.

**Conclusion:**

Non-medical barriers in GI cancer care undermine patient care, provider capacity, and health system efficiency. These findings provide an actionable framework for developing equitable, efficient, and sustainable cancer care models.

**Supplementary Information:**

The online version contains supplementary material available at 10.1007/s00520-026-10897-3.

## Introduction

Modern treatment of gastrointestinal (GI) cancers is resource-intensive and requires coordination among surgical, medical, and radiation oncology, as well as support from nutrition, social work, psychology, and rehabilitation services, each with distinct workflows [1]. Prior studies show that these cross-specialty transitions are frequent sites of care disruption, contributing to delays in diagnosis, treatment initiation, and survivorship planning [2–4]. These vulnerabilities are amplified when patients encounter non-medical barriers, including inadequate insurance coverage, limited transportation, or low health literacy, which disproportionately affect socioeconomically disadvantaged or racially minoritized populations [5–7]. National studies indicate that these barriers are common in GI cancer care, with approximately 45% of patients experiencing financial toxicity and 28% reporting at least one health-related social risk [8]. In response, our institution is developing a transdisciplinary care model to address non-medical determinants of care completion through proactive navigation, provider-delivered education, and resource coordination [9].

To inform this effort, we previously surveyed providers across multiple disciplines at our institution to identify perceived barriers to GI cancer care [10]. Providers most frequently cited insurance and health literacy barriers during initial treatment planning and reported that navigating these challenges consumed a substantial portion of their workday. These findings are consistent with national data showing that while 93% of oncologists recognize the impact of social determinants on outcomes, 81% report having limited time to address patients’ social needs [11]. The downstream consequences are significant: administrative and coordination burdens have been identified as key drivers of oncology clinician burnout and reduced system efficiency [12, 13]. However, while the relationship between administrative burden and burnout is increasingly documented, the specific mechanisms by which patient non-medical barriers generate workflow disruption across multidisciplinary teams remain poorly understood. Existing qualitative work has explored provider perspectives on social determinants in oncology, but has focused on single disciplines, single barrier domains, or non-GI settings [14, 15]. Our survey data highlighted the scope of this problem but did not elucidate the mechanisms by which non-medical barriers generate these downstream effects—a gap that qualitative methods are uniquely positioned to address [16].

Accordingly, we conducted a qualitative descriptive study using semi-structured interviews with multidisciplinary providers caring for patients with GI cancers. We examined how providers across roles and settings perceive and navigate non-medical barriers, how these barriers shape workflow and capacity, and the strategies envisioned to improve coordination and equity. By purposively sampling across disciplines, this study addresses a gap in the existing qualitative literature on non-medical barriers in GI cancer care.

## Methods

### Study design

This qualitative descriptive study constitutes the second phase of a multidisciplinary project assessing provider perspectives on non-medical barriers to GI cancer care and identifying system-level solutions. Semi-structured interviews elicited providers’ experiences with patient- and system-level barriers, workflow challenges, and opportunities to improve engagement and coordination. A brief Research Electronic Data Capture (REDCap) [17] survey characterized participant demographics and practice context.

The study was approved by the University Hospitals Institutional Review Board (STUDY20231174). Reporting follows the Consolidated Criteria for Reporting Qualitative Research (COREQ) guidelines [18] (Supplementary Document 1).

### Sample and recruitment

We used purposive sampling to recruit healthcare providers directly involved in GI cancer care at University Hospitals Seidman Cancer Center, a National Cancer Institute (NCI)-designated comprehensive cancer center serving a socioeconomically and racially diverse 15-county catchment in Northeast Ohio. Eligible participants included physicians, advanced practice providers (APPs), nurses, dietitians, social workers, care coordinators, and rehabilitation therapists. Recruitment occurred through direct outreach. Email invitations described the study’s purpose, voluntary nature, and assurances of confidentiality. Interested providers contacted the study team to schedule interviews. All participants provided informed consent and received no incentives.

Given the exploratory qualitative design, a formal a priori sample size calculation was not performed. Recruitment and analysis proceeded iteratively until thematic saturation was reached, defined as the point at which additional interviews yielded no substantively new codes or themes.

### Setting and participants

Interviews were conducted virtually via Zoom by trained study personnel (T.L.) between March and July of 2025. T.L. is a female resident physician (MD) who is trained in qualitative research. Some providers knew the interviewer as a trainee within the hospital system. Only the participant and the interviewer were present. Participants were drawn from inpatient and outpatient settings, with varying years of experience.

### Survey instrument

The survey collected participant demographics and practice characteristics, including gender, age, race and ethnicity, credentials, primary role, years in role and at the institution, employment status (full- or part-time), percentage of GI cancer patients in their practice, and the number of practice sites. All items were closed-ended. The complete instrument is provided in Supplementary Document 2.

### Interview guide

The semi-structured interview guide was developed collaboratively by the investigative team and informed by the prior provider survey and the Consolidated Framework for Implementation Research (CFIR) [10, 19] (Supplementary Document 3). Questions explored how non-medical barriers manifest in practice, how providers navigate them, and strategies to improve coordination and equity. Each domain included open-ended prompts with neutral probes to elicit detailed narratives while allowing flexibility to pursue emergent themes. The guide concluded with appreciative inquiry-based questions to identify strengths and feasible multidisciplinary solutions [20]. Interviews were designed to last approximately 30 min. The first five interviews served as pilots. The guide was refined for clarity and probe depth following these interviews, with no structural changes made to core questions, and all five were retained in the final analysis.

### Data collection

Interviews lasted approximately 20–30 min and were audio-recorded on Zoom. Transcripts were generated using Zoom’s artificial intelligence software, reviewed for accuracy, and de-identified. Data collection and early analysis occurred concurrently to allow iterative refinement of the interview guide as themes emerged. No repeat interviews were conducted, and transcripts were not returned to participants, as the focus was descriptive rather than interpretive.

### Data analysis

The analytic objective was to characterize how non-medical barriers are perceived and navigated across roles and settings and to derive actionable themes to inform system-level interventions. Survey variables were summarized descriptively (counts and percentages for categorical items; medians with interquartile ranges for continuous items).

Transcripts were inductively analyzed by two coders (D.M. and R.C.) using Braun and Clarke’s reflexive thematic analysis framework [21]. Coding was performed in NVivo version 14 using a structured codebook to enhance consistency. Inter-rater reliability for subcodes was assessed using Cohen’s *κ*, summarized as mean and median values, and categorized per McHugh’s criteria (none, minimal, weak, moderate, strong, almost perfect) [22]. Of note, findings with low inter-rater reliability should be interpreted with caution, as *κ *is sensitive to code prevalence and rater bias and may produce paradoxically low values for infrequent codes even when raw percent agreement is high [23]. Both coders developed and piloted a shared codebook by double-coding a subset of transcripts, and then resolved discrepancies through structured consensus discussion facilitated by the senior author (T.L.), with iterative codebook refinement targeting moderate agreement (*κ* ≥ 0.40) across subthemes prior to coding the remaining transcripts.

Pearson correlation coefficients (*r*) between parent themes were calculated using interview-level reference counts (one vector per theme across 30 interviews). Correlations informed node classification and edge selection, with *r* values greater than 0.70 considered high and those greater than 0.85 considered very strong. Correlations were used to triangulate qualitative inferences and were not interpreted as causal.

### Directed acyclic graph

To examine hierarchical relationships among barriers, a directed acyclic graph (DAG) was constructed as a conceptual, hypothesis-generating framework informed by epidemiologic causal inference methodology [24, 25]. Transcripts were reviewed for directional language (e.g., “causes,” “leads to,” “because,” “when X then Y”), and statements were mapped by theme to identify hypothesized directional pathways.

Barriers were classified as upstream structural factors (analogous to true confounders), intermediate variables that transmit upstream effects (proxy confounders), or proximal factors that may directly shape outcomes (exposures/mediators). Classification drew on three converging lines of evidence: (1) correlation strengths among coded subthemes, (2) co-occurrence patterns across interviews, and (3) directional language from provider narratives. Themes were designated as upstream structural factors if they demonstrated a correlation coefficient ≥ 0.80 with multiple other barrier domains and were recurrently described by providers as antecedent conditions. These thresholds were selected a priori to prioritize the most robust co-occurrence patterns and should be interpreted as conservative, hypothesis-generating benchmarks rather than causal criteria. Only the strongest hypothesized pathways (*r* ≥ 0.78) were retained in the final DAG. Correlations were used to triangulate qualitative inferences and were not interpreted as causal; the resulting model represents a conceptual framework to guide future confirmatory research.

### Rigor and trustworthiness

We enhanced rigor through double coding, an audit trail, reflexive memos, and biweekly debriefings with T.L., supported by detailed contextual description and triangulation across survey data, interviews, and visual mapping.

## Results

### Participant characteristics

Thirty healthcare providers participated in semi-structured interviews, representing a multidisciplinary sample involved in GI cancer care. Of the 32 invited, two declined participation. Twenty-eight participants completed the accompanying survey on provider demographics and practice characteristics (Table 1).

**Table 1:** Participant demographics and practice characteristics

Characteristic	*n*^a^ (%)/median (IQR)
Gender	
Female	24 (85.7%)
Male	4 (14.3%)
Age	
Age, years	45 (33.8–53.5)
Race/ethnicity	
Hispanic/Latino origin	3 (10.7%)
White	25 (89.3%)
Black or African American	1 (3.6%)
Multi-racial	1 (3.6%)
Prefer not to answer	1 (3.6%)
Primary role	
Physician (MD/DO)	7 (25.0%)
Dietitian	7 (25.0%)
Social worker	6 (21.4%)
Nurse (RN)	5 (17.9%)
Advanced practice provider	2 (7.1%)
Care coordinator	1 (3.6%)
Experience	
Full-time employee	24 (85.7%)
Years in current role	9 (3.5–14.8)
Years at institution	10 (4.1–16.3)
Estimated % GI caseload	65 (40–90)
Number of sites covered	1 (1–2)
Number of institutions worked at in last 5 years	1 (1–2)

^a^Demographics reflect *n* = 28 participants who completed the accompanying survey; two participants completed interviews but did not complete the survey and are excluded from this table

Most respondents identified as female (85.7%) and White (89.3%), with a median age of 45 years (IQR, 33.8–53.5). Participants represented diverse professional roles, including physicians (25.0%), dietitians (25.0%), social workers (21.4%), nurses (17.9%), APPs (7.1%), and care coordinators (3.6%). Most were full-time employees (85.7%), with a median of 9 years in their current role and a median GI oncology caseload of 65% (IQR, 40–90). Participants practiced in both inpatient and outpatient settings.

### Identified themes

Thematic analysis identified seven major themes encompassing 19 subthemes: (1) health system barriers; (2) financial barriers; (3) access and logistics; (4) provider capacity; (5) patient knowledge and engagement; (6) social and emotional barriers; and (7) communication and coordination (Table 2). Themes reflected multilevel, interconnected institutional and patient-level challenges.

**Table 2:** Themes, subthemes, and exemplar quotations from semi-structured provider interviews

Theme	Exemplary quote
Theme 1: Health system barriers	
Care navigation	“We’ve sent referrals to 21 facilities, and nobody will take him with TPN. Like he doesn’t need to be in the hospital, but that’s also like then, poor quality for the patient. He’s getting depressed.” (Participant 2, advanced practice provider)
Protocols and systems	“When you’re just constantly having to start over, it’s just exhausting.” (Participant 2, advanced practice provider)
Staffing and turnover	“I’ve been out in the Community Center for gosh, like 5 or 6 years now, and I know there’s been a lot of turnover in our inpatient side… communication just isn’t maybe as great… as it could be.” (Participant 9, dietitian)
Theme 2: Financial barriers	
Insurance gaps	“Patients who need pancreatic enzymes especially patients with Medicare have a significant copay for the enzymes and oftentimes it’s thousands of dollars a month. I think the highest copay I heard was $6,000 a month.” (Participant 9, dietitian)
Care cost	“A lot of people are taking time off of work… Some people are driving 30, 45 min to come in, and that’s a lot of gas money for them… And then there’s the cost of just you know the medications… most patients that’s just a lot of money for them to spend. In addition to trying to be able to have food.” (Participant 19, physician)
Income loss	“She could take FMLA, but she can’t. They don’t have short term disability benefits. Well, guess what FMLA doesn’t pay, and she needs to support her 3 children. She’s a single mother.” (Participant 17, social worker)
Financial navigation	“If the patient isn’t approved you know, if they their income level, you know, just above, you know, whatever criteria that’s been set, you know, they may still be stuck with, you know, several $1,000 Copay that they are unable to afford.” (Participant 9, dietitian)
Theme 3: Access and logistics	
Transportation	“Transportation is always a barrier to getting people to appointments. Some folks have a transportation benefit as a part of their health insurance. Those transportation options are not always the most reliable. They’re often late, sometimes they don’t show up at all.” (Participant 20, social worker)
	“We’ve actually had to coordinate, using our own resources, 2 Ubers once here and once back every day for 7 weeks. We’re spending approximately $1,500 just on this one patient… But without the round trip option he would essentially not be able to receive treatment whatsoever.” (Participant 23, social worker)
Geography	“If the patient is elderly or they’re not able to drive to their appointment, it’s been difficult for them, especially if they need more specialized care and have to go up to the main campus.” (Participant 14, dietitian)
Placement issues	“The nursing facility has to take on the burden of the cost of that medication. And if that medication costs more than they’re going to charge that patient today, they’re not going to take that patient.” (Participant 5, social worker)
Theme 4: Provider capacity	
Time constraints	“About 25 to 30% of my time is spent addressing barriers, and that does [feel like a lot], especially when we have such a large patient population.” (Participant 6, care coordinator)
	“It forces you to reprioritize the day for sure, reorganize the day… It just takes away from that direct [care].” (Participant 2, advanced practice provider)
Provider burnout	“Oh, yeah, absolutely [it contributes to burnout]… when you know a patient can do better overall, like they have a better quality of life if these services were available to them, and to see them discharge or go home without these services. It’s almost feeling like we were defeated that day.” (Participant 6, care coordinator)
Theme 5: Patient knowledge and engagement	
Health literacy	“He had a gastrectomy… Then he came back 2 weeks later and had to be admitted due to acute renal failure. He was extremely dehydrated and malnourished… the granddaughter told me her grandfather acts like he knows what we’re telling him, but doesn’t… I felt really bad because I did spend time with him, and I thought the plan was doable, and it completely fell through.” (Participant 8, dietitian)
Education gaps	“Some, even when they have decent health literacy are still unprepared and have not had enough education like preoperatively beforehand, and when they end up being on the floor are like overwhelmed with everything that’s going on… the brunt of their like expectations and things that they want are like directed towards the nurse, and when it’s not been like previously explained to them what they need, it kind of falls upon us sometimes to try and educate them when it’s not always within our scope.” (Participant 3, nurse)
Patient activation	“We do have education classes. I know the teach back method and all that, but you know, sometimes again, it’s just people parroting what you want they think you want to hear, instead of what’s actually going to happen.” (Participant 8, dietitian)
Theme 6: Social and emotional barriers	
Social support	“Some patients live on their own and, you know, if they’re not feeling well, it can be harder to care for themselves. And, you know, that translates into like going for groceries and again, maybe just not feeling up to making foods, purchasing foods.” (Participant 9, dietitian)
	“I have plenty of patients that don’t have any support except for themselves. And so when they lose that job, or they don’t have enough money coming in from the job, even if they’re working full time, it’s just not enough.” (Participant 19, physician)
Emotional burden	“Sometimes, they don’t want the support because they’re embarrassed, because, you know, they saw this coming, especially with colon cancer. You know they saw the signs and they didn’t say anything.” (Participant 17, social worker)
Theme 7: Communication and coordination	
Team communication	“Having open communication with one another. And being able to talk to that person versus trying to communicate through email, or, you know, secure messaging is really helpful… [But] communication just isn’t maybe as great as it could be [between inpatient and outpatient].” (Participant 9, dietitian)
Discharge transitions	“I have talked to a lot of our social workers about like issues with doctors, not communicating to them properly… upon like discharges, and they end up like having to like, scramble to try and solve all the issues.” (Participant 3, nurse)

**Fig. 1:**
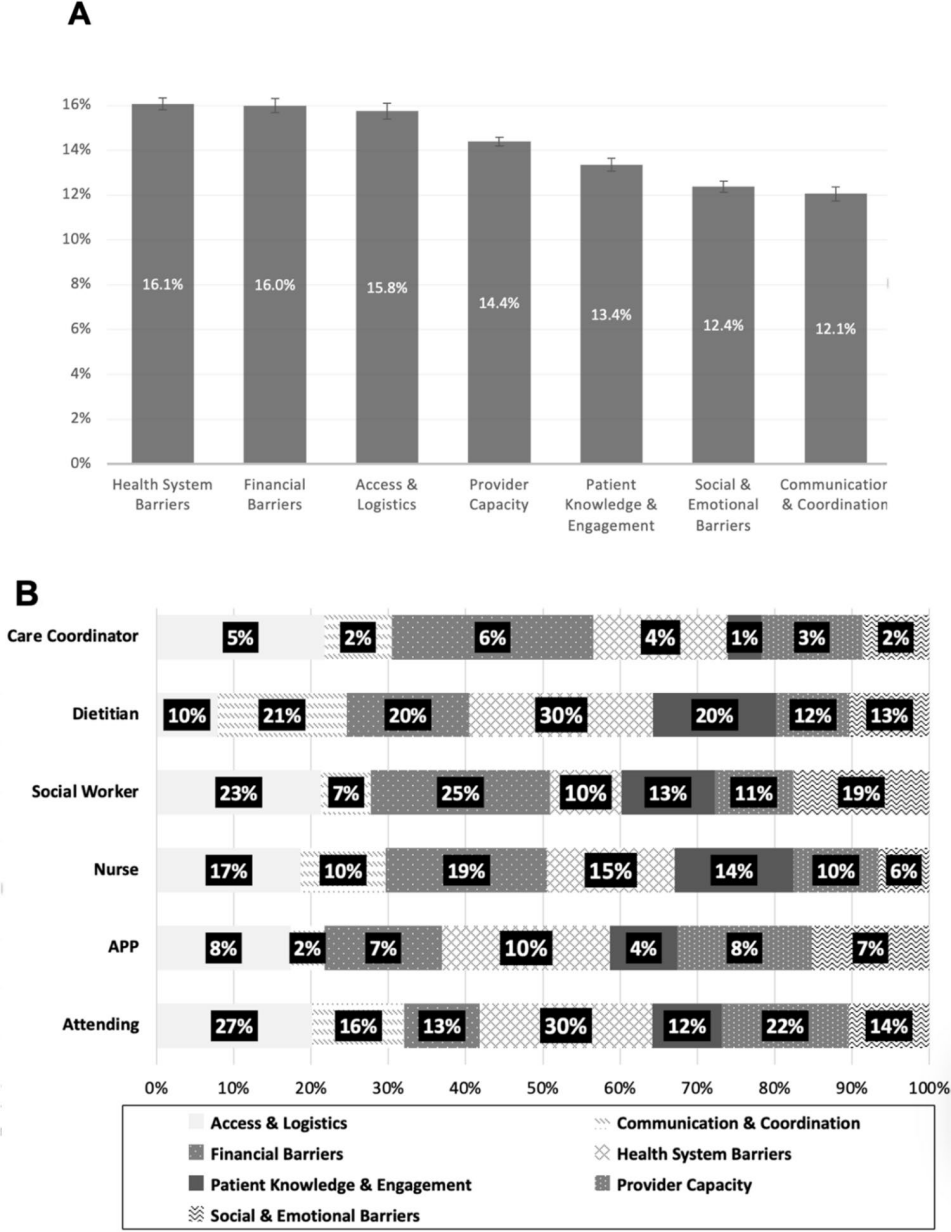
Thematic prevalence and role-specific composition in provider interviews. **A** Mean proportion of coded references per interview for each theme (*n* = 30 interviews). Error bars show 95% confidence intervals. Themes are ordered from most to least prevalent. **B** Within-role composition of coded content across seven themes. Stacked bars sum to 100% within role (attending *n* = 7, APP *n* = 2, nurse *n* = 5, social worker *n* = 6, dietitian *n* = 7, care coordinator *n* = 1). APP, advanced practice provider

Figure 1A illustrates the relative frequency of each theme, with modest variability across interviews (standard deviation ~ 3%), suggesting broad relevance across roles. The most prevalent themes were health system barriers (16.07%), financial barriers (16.00%), and access and logistics (15.75%).

Analysis by provider type reveals distinct disciplinary emphases (Fig. 1B). Nurses, social workers, and care coordinators most frequently discussed financial barriers, while attendings, APPs, and dietitians emphasized patient knowledge and engagement. Health system barriers were most prominent among dietitians and care coordinators and least represented among social workers. Attendings and APPs most frequently discussed provider capacity, and dietitians most frequently discussed communication and coordination barriers.

#### Theme 1: Health system barriers

Providers described systemic barriers, such as fragmented navigation, inconsistent protocols, and workforce turnover, that delayed care and strained coordination. Subthemes included care navigation; protocols and systems; and staffing and turnover. Unclear pathways often left patients “stuck” in the system, particularly when discharge planning required cross-facility coordination: “We’ve sent referrals to 21 facilities, and nobody will take him with TPN… he doesn’t need to be in the hospital” (participant 2, APP). Providers emphasized that authorization requirements and staffing instability compounded inefficiency and undermined continuity.

#### Theme 2: Financial barriers

Economic constraints shaped care across the continuum through insurance gaps, care costs, income loss, and financial navigation. Providers described cumulative financial toxicity as a common cause of delayed or incomplete therapy: “A lot of people are taking time off of work… Some people are driving 30, 45 [minutes]… and that’s a lot of gas money for them… And then there’s the cost of… the medications… that’s just a lot of money… in addition to trying to be able to have food” (participant 19, physician).

#### Theme 3: Access and logistics

Access barriers, including transportation, geography, and placement issues, often became the rate-limiting step even when treatment plans were clinically appropriate. Transportation was described as near-universal, sometimes requiring extensive coordination to prevent missed therapy: “We’ve actually had to coordinate, using our own resources, 2 Ubers once here and once back every day for 7 weeks. We’re spending approximately $1,500 just on this one patient… But without [this], he would essentially not be able to receive treatment whatsoever” (participant 23, social worker).

#### Theme 4: Provider capacity

Providers reported that addressing non-medical barriers consumed time and emotional energy, contributing to burnout and moral distress (time constraints and provider burnout): “When you know a patient can do better overall, like they have a better quality of life if these services were available to them, and to see them discharge or go home without these services. It’s almost feeling like we were defeated that day” (participant 6, care coordinator).

In contrast to other provider types, most (five out of six) social workers described this work as aligned with their scope and, at times, a source of job satisfaction. One provider stated, “I think it adds to job satisfaction. I learned a long time ago that that we can’t fix everything and I’ve learned, too, that patients appreciate that we try to help them… I think that’s kind of been my mindset, like we’re doing the best we can to help the patients. And while it’s frustrating that the systems don’t always cooperate with us, I think that knowing that I’m trying to help the patient… find new avenues or… think about things in a different way…That’s why I’m doing what I’m doing” (participant 21, social worker).

#### Theme 5: Patient knowledge and engagement

Providers described health literacy, education gaps, and patient activation as persistent barriers, particularly around complex treatment plans and transitions. As one clinician noted, “Some, even when they have decent health literacy, are still unprepared and have not had enough education… preoperatively” (participant 3, nurse). Another observed that standard approaches can fail to translate into action: “Sometimes again, it’s just people parroting what you want they think you want to hear, instead of what’s actually going to happen” (participant 8, dietitian).

#### Theme 6: Social and emotional barriers

Social isolation and emotional distress (social support; emotional burden) undermined patients’ ability to sustain treatment. Providers noted that embarrassment, fear, and limited support could delay disclosure of symptoms and engagement with care: “They don’t want the support because they’re embarrassed” (participant 17, social worker). Providers described a bidirectional burden, with patients lacking support and clinicians absorbing emotional strain.

#### Theme 7: Communication and coordination

Fragmented inpatient-outpatient communication contributed to duplicative work and weak continuity (team communication; discharge transitions): “Communication just isn’t maybe as great as it could be” (participant 9, dietitian). Participants emphasized earlier social work engagement and clearer handoffs as opportunities to reduce delays and prevent readmissions.

### Interrater reliability

Interrater reliability across 19 subthemes was moderate (mean *κ* = 0.53), with variation across codes (Table 3). Using McHugh’s criteria, agreement ranged from none/minimal to strong; 8 of 19 subthemes met *κ* ≥ 0.60, including protocols and systems, care costs, income loss, geography, placement issues, education gaps, emotional burden, and discharge transitions.

**Table 3:** Interrater agreement for thematic subcodes

Subtheme	Mean kappa	Median kappa	McHugh category
Health system barriers			
Care navigation	0.528	0.555	Weak
Protocols and systems	0.682	0.864	Strong
Staffing and turnover	0.139	0.000	None
Financial barriers			
Care costs	0.605	0.615	Moderate
Financial navigation	0.250	0.000	None
Income loss	0.794	0.829	Strong
Insurance gaps	0.216	0.250	Minimal
Access and logistics			
Geography	0.636	0.671	Moderate
Placement issues	0.614	0.727	Moderate
Transportation	0.181	0.200	Minimal
Provider capacity			
Provider burnout	0.329	0.302	Minimal
Time constraints	0.330	0.156	Minimal
Patient knowledge and engagement			
education gaps	0.766	0.783	Moderate
health literacy	0.568	0.635	Weak
patient activation	0.500	0.500	Weak
Social and emotional barriers			
Emotional burden	0.608	0.715	Moderate
Social support	0.462	0.462	Weak
Communication and coordination			
Discharge transitions	0.748	1.000	Moderate
Team communication	0.250	0.000	Weak

### Directed acyclic graph and causal pathways

**Fig. 2:**
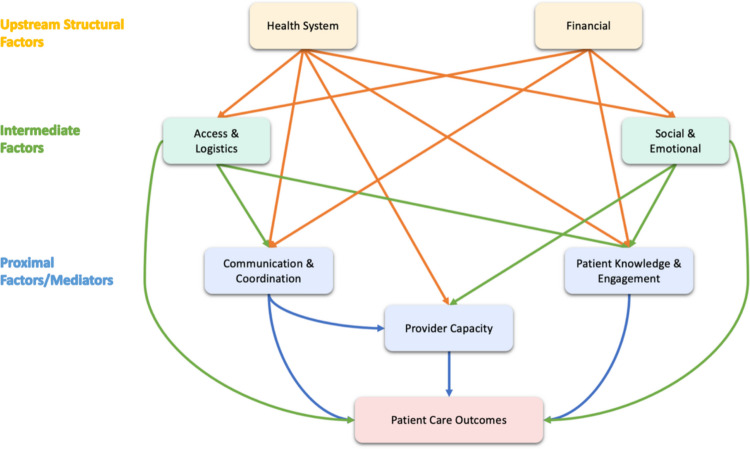
Conceptual framework of hypothesized relationships among non-medical barrier domains. Hypothesis-generating diagram illustrating hypothesized directional relationships among interview-derived barrier domains. Orange = upstream structural factors (Health System, Financial); green = intermediate factors (access and logistics, social and emotional); blue = proximal factors/mediators (communication and coordination, patient knowledge and engagement) hypothesized to act through provider capacity to influence patient care outcomes. Arrows represent hypothesized pathways derived from provider narratives and thematic co-occurrence; they do not imply confirmed causal relationships

The DAG illustrated hypothesized directional relationships among the seven barrier domains as a conceptual, hypothesis-generating framework (Fig. 2). Health system and financial barriers emerged as upstream structural factors with the broadest co-occurrence across domains. Health system barriers strongly correlated with communication and coordination; patient knowledge and engagement; provider capacity; access and logistics; and social and emotional barriers (all *r* > 0.81). As one provider noted, “We lost our dedicated schedulers… now 50% of appointments aren’t scheduled correctly, so I check all orders myself, and patient care gets delayed” (participant 25, nurse). Financial barriers similarly correlated with communication and coordination; provider capacity; and patient knowledge and engagement (all *r *> 0.82).

Social and emotional barriers and access and logistics functioned as intermediate factors, hypothesized to transmit the effects of upstream structural barriers to workflow-level challenges. Access and logistics correlated most strongly with financial barriers (*r* = 0.85), consistent with provider narratives describing how transportation costs and geographic distance contributed to missed educational opportunities and coordination breakdowns. Provider capacity emerged as a central convergence point, with co-occurrence patterns and provider narratives suggesting associations with both health system barriers and social and emotional barriers. Failures across these domains were associated with patient outcomes, including treatment delays and preventable readmissions.

### Perceived solutions and transdisciplinary integration

When a transdisciplinary care model was introduced, participants consistently described it as potentially beneficial for patients, providers, and care teams. Providers emphasized early integration of social work, nutrition, and financial counseling, with clear role delineation and a single point of contact. Several highlighted the need for a designated coordinator to streamline referrals and communication. Three participants cautioned that new structures should not become “another thing we need to do” (participant 1, dietitian). In contrast, another noted, “even though it would be extra time to have those meetings, it would probably save time in the long run, because everyone would be there to be able to help the patient the best way they can” (participant 7, dietitian) (Table 4).

**Table 4:** Provider perspectives on the transdisciplinary team model by question

Question	Answer
How might this help your patients?	I would say, even though it would be extra time to have those meetings, it would probably save time in the long run, because everyone would be there to be able to help the patient the best way they can. (Participant 7, dietitian)
	I think you know, having that comprehensive approach to care would be ideal for cancer patients… and I think that would be a great team approach, and I’m sure that disciplines would… feel comfortable with each other, and be able to communicate well within the group… that would be awesome. (Participant 9, dietitian)
	My patients… feel that care is disconnected even among the doctors. They have one say one thing, one say another thing, and it’s going back and getting clarification. So I think any time that you’re putting everybody in a room together and coming up with a plan is beyond important. It gives the patients confidence that we know what we’re doing versus I don’t really know why they told you two different things… I know these patients are already scared, so if they even lose a little bit of confidence in us, we’re doing them a disservice, so I would absolutely love it. (Participant 12, dietitian)
How might it support you and your team?	I think my only thing would be… I don’t want it to be seen as if it’s… another thing we need to do, because I think that [contributes to] burnout. (Participant 1, dietitian)
	I think if there was a single point of contact within that group that my nurse partner could reach out to as issues arise that would certainly streamline the process… so that we don’t have to make a separate call to the dieticians, and a separate call to the financial counselor, one person, one stop shopping to do it all. (Participant 4, physician)
	It would give me confidence that people are not being lost within the shuffle. (Participant 22, physician)
If you were designing this program, what would be the most important elements?	But I just know having someone within the healthcare system just with who’s part of the team helping them make feel supported that things aren’t falling through that can follow up, and I think that would just really help them, so I think a patient navigator is just critical to having a successful team. (Participant 19, physician)
	Having the patient prioritize what’s important to them. (Participant 6, care coordinator)
	Social worker definitely. Having a key person that can kind of gather that information to direct them to the right thing, I mean, sometimes I think referrals are sent in, but you know they don’t really know how to navigate that system. (Participant 10, nurse)

## Discussion

The present study evaluating provider perspectives on non-medical barriers to GI cancer care identified three principal findings. First, providers described multilevel, interconnected barriers spanning health system, financial, logistical, and psychological domains that constrain timely and equitable care delivery. Second, these barriers extended beyond patient hardship to reduce provider capacity, contributing to inefficiency, burnout, and moral distress, particularly among clinicians who view such tasks as outside their professional role. Third, providers expressed support for a transdisciplinary care model to coordinate resources, enhance the patient experience, and alleviate provider strain. Collectively, these findings suggest that non-medical barriers function not as isolated patient-level challenges, but as system-wide forces that erode equity, quality, and workforce sustainability, reinforcing the need for coordinated solutions.

Of the seven themes identified, health system, financial, and access and logistics barriers were most prominent, aligning with prior studies describing multilevel obstacles to cancer care delivery [26–28]. Our data extend this work by clarifying how social and structural constraints may interact to generate clinical inefficiency. As illustrated in the conceptual framework, health system and financial barriers were hypothesized to be upstream structural factors associated with downstream processes, including institutional capacity, workflow, and access to care. Access and logistics and social and emotional barriers appeared to translate these pressures into practical constraints, including transportation challenges, geographic distance, and limited social support. These, in turn, were associated with communication and coordination; provider capacity; and patient knowledge and engagement—domains where structural strain may most directly influence patient outcomes. This pattern suggests that delays, fragmentation, and inequities in GI cancer care may arise from cumulative, cascading interactions between system design, workforce strain, and resource scarcity rather than discrete failures, though this hypothesis warrants confirmation in future research. Addressing these barriers, therefore, requires multilevel interventions targeting both structural roots and proximal mechanisms, including communication, education, and provider support.

Provider interviews further clarified how non-medical barriers reverberate through the workforce, reducing capacity and increasing moral distress. Participants described the persistent effort required to address transportation challenges, insurance denials, and communication gaps as diverting time and attention from direct patient care. Consistent with prior qualitative studies, misalignment between assigned responsibilities and actual practice emerged as a key contributor to frustration and burnout [29, 30]. However, this burden was unevenly distributed across disciplines. For social workers, whose roles explicitly encompass navigation and psychosocial support, such tasks aligned with professional scope and were less likely to induce strain. In contrast, clinicians and dietitians viewed these responsibilities as outside their role, amplifying distress. Prior research indicates that organizational interventions, such as adding support staff and streamlining workflows, are most effective in reducing burnout and improving well-being [31]. Together, these insights highlight the importance of aligning task allocation and team design with professional roles to address non-medical needs without compromising clinician capacity.

Across disciplines, providers articulated strong support for a transdisciplinary care model to mitigate these interconnected barriers. They emphasized the potential of such a structure to restore continuity, reduce patient confusion, and strengthen interprofessional trust by addressing persistent communication gaps. Prior studies note that the cumulative cost of staff time and scheduling regular meetings, particularly for specialists covering multiple tumor types or sites, can limit participation [32–34]. Providers in the present study echoed these concerns, underscoring the importance of integrating any new structure into existing workflows rather than adding burden. Evidence from other settings demonstrates that sustained organizational commitment, leadership engagement, flexibility for local adaptation, and iterative feedback are critical for successful implementation [34, 35]. In this context, provider recommendations—such as establishing a single point of contact, clearly delineated roles, and integrating navigation and social services early—offer practical guidance for designing scalable, sustainable models that support patients and care teams alike.

This study should be interpreted in light of several limitations. First, it was conducted at a single NCI-designated comprehensive cancer center with dedicated navigation resources that may not be available at community or safety-net hospitals; barriers identified here may be even more pronounced in low-resource settings. Findings are best understood as analytically transferable rather than generalizable. Second, voluntary recruitment via email may have preferentially enrolled providers more attuned to systemic barriers, potentially amplifying the salience of certain themes. Third, although transcripts were double-coded and underwent iterative consensus review targeting moderate agreement, inter-rater reliability varied across subthemes. Mean *κ* was 0.53, consistent with moderate agreement per McHugh’s criteria [22]; however, several subthemes fell in the minimal-to-weak range, which may reflect inherent interpretive ambiguity and the statistical sensitivity of* κ* to low-prevalence codes [23]. Findings from lower-*κ* subthemes should be interpreted with appropriate caution. Fourth, the DAG should be interpreted as a conceptual, hypothesis-generating model; causal inferences cannot be drawn from qualitative co-occurrence and provider narrative alone, particularly given the purposive sample of 30 providers from a single health system. The correlation thresholds used to define pathways require validation in future confirmatory research. Finally, as this phase of the study captured only provider perspectives, triangulation with patient and caregiver interviews (currently underway) will be essential to fully characterize the multilevel factors influencing care delivery.

## Conclusion

Building on our initial provider survey [10], these interviews deepen our understanding of how structural, financial, and logistical barriers intersect to shape both patient outcomes and provider capacity. Together with forthcoming patient and caregiver perspectives, these findings will directly inform the development and implementation of a multilevel, transdisciplinary, equity-focused team to support our most vulnerable GI cancer patients. The implications of this work extend across levels of care. For patients, the findings highlight the need for integrated navigation, education, and resource coordination to improve access and equity. For providers, they emphasize the importance of team-based models that clarify roles, reduce administrative burdens, and enhance communication to promote clinician well-being. For health systems and policymakers, the emphasis is on leadership engagement, sustainable infrastructure, and funding mechanisms that support coordinated care. Collectively, this work moves beyond identifying barriers to building a practical, scalable framework that embeds equity and efficiency into the fabric of cancer care delivery.

## Supplementary Information

Below is the link to the electronic supplementary material.

Supplementary file1 (PDF 369 KB)

Supplementary file2 (PDF 36.7 KB)

Supplementary file3 (DOCX 24.3 KB)

## Data Availability

The qualitative interview data are not available for sharing because participants did not provide consent for transcript sharing beyond the study team and because of the risk of re-identification. Additional materials (e.g., interview guide and codebook excerpts) may be available from the corresponding author upon reasonable request.
