# Supplementary material for: Beyond the patient: how providers perceive and experience non-medical barriers in gastrointestinal cancer care
Source: Support Care Cancer. 2026 Jun 18;34(7):671. doi: 10.1007/s00520-026-10897-3 (PMC13279395; doi:10.1007/s00520-026-10897-3)
Supplement: Supplementary file 2 — Supplementary file2 (PDF 36.7 KB) [file 520_2026_10897_MOESM2_ESM.pdf]

Provider Demographics

Please complete the survey below before the interview.

Thank you!

What is your gender?	◯ Male<br>◯ Female<br>◯ Non-binary<br>◯ Prefer not to say
What is your age?	__________________________
Are you of Hispanic, Latino, or Spanish origin?	◯ Yes<br>◯ No<br>◯ Prefer not to answer
What is your race/ethnicity? (Select all that apply)	☐ American Indian or Alaska Native<br>☐ Asian<br>☐ Black or African American<br>☐ Native Hawaiian or Other Pacific Islander<br>☐ White<br>☐ Prefer not to answer
What is your degree(s)? (Check all that apply)	☐ MD/DO<br>☐ PA (Physician Assistant)<br>☐ NP (Nurse Practitioner)<br>☐ RN (Registered Nurse)<br>☐ LCSW (Licensed Clinical Social Worker)<br>☐ PhD<br>☐ Other (please specify)
If you selected other in the previous question, please specify.	__________________________
What is your primary role in healthcare?	◯ Physician<br>◯ Advanced Practice Provider (APP)<br>◯ Nurse<br>◯ Social Worker<br>◯ Care Coordinator<br>◯ Physical/Occupational Therapist<br>◯ Dietitian<br>◯ Other (please specify)
If you selected other in the previous question, please specify.	__________________________
How many years have you been practicing in your current role?	__________________________
How many years have you worked at University Hospitals?	__________________________
Approximately what percentage of your patients are diagnosed with gastrointestinal (GI) cancers?	__________________________
How many UH facilities do you currently work at?	__________________________

How many different healthcare institutions have you worked at in the past 5 years?	__________________________________
What is your current employment status?	◯ Full-time<br>◯ Part-time<br>◯ As needed
