# Supplementary material for: Beyond the patient: how providers perceive and experience non-medical barriers in gastrointestinal cancer care
Source: Support Care Cancer. 2026 Jun 18;34(7):671. doi: 10.1007/s00520-026-10897-3 (PMC13279395; doi:10.1007/s00520-026-10897-3)
Supplement: Supplementary file 3 — Supplementary file3 (DOCX 24.3 KB) [file 520_2026_10897_MOESM3_ESM.docx]

**Provider Perspectives on Barriers to GI Cancer Care: Semi-Structured Interview Guide**

**Aims:**

- Contextualize provider perspectives on barriers and facilitators to care for patients with gastrointestinal (GI) cancers.
- Understand the provider’s role and the coordination required to mitigate care disparities.
- Identify actionable strategies and resources to address non-medical barriers to care.

**Structure:**

The interviews will be semi-structured, with open-ended questions that can be adapted to reflect the provided responses. The conversation should explore issues as they are raised, with guidance and prompting as required. Interviewers will ensure the conversation progresses efficiently and remains relevant to the study's aims. Interviews are expected to last approximately 30 minutes.

**Content:**

The interviewer will first review the aims of the session and the participant information sheet, emphasizing confidentiality, consent, privacy, and the right not to participate. Virtual consent will be obtained before recording the session. The content of each interview will be based on (a) the results of the Phase 1 survey and (b) the Consolidated Framework for Implementation Research.

**Introduction**

Thank you for talking with me today. We are interested in learning more about providers’ experiences caring for patients with gastrointestinal (GI) cancers, particularly the challenges you encounter and how you and your team work to overcome those challenges.

I will be asking you some questions about **your role as a provider**, the **barriers and facilitators you see when caring for GI cancer patients**, and **your thoughts on how we can better support patients facing non-medical challenges**. We are especially interested in understanding where systems or processes could be improved, particularly to help patients with fewer resources or more complex needs.

You will notice that I will not give you much feedback on your responses because I do not want to influence your answers. Please know there are no right or wrong answers – we are here to learn from you.

Finally, you are not obligated to discuss anything you are uncomfortable discussing with me. Do you have any questions or concerns before we begin?

We will now move to the interview. I will start the recording now.

Today’s date is: [insert date], and I am interviewing participant [use study ID].

Today, I will refer to you as your ID number, not your name, for confidentiality purposes. Please remember not to use any specific names as well.

**Section 1. Contextualize provider perspectives on barriers and facilitators to care for gastrointestinal (GI) cancer patients.**

I want to start by learning a bit about you and your experience with GI cancer care.

Could you tell me a bit about your current role?

*(Probes: How long have you been in this role? What are some of your responsibilities? How frequently do you work with GI cancer patients?)*

We want to hear **your perspective on the non-medical barriers to care that GI cancer patients face**.

From your perspective, what are the **biggest challenges these patients face** when trying to get the care they need, especially those with fewer resources or support systems?

- Insurance
- Food insecurity
- Geographic location
- Transportation difficulties
- Employment and work-related issues
- Housing instability
- Lack of social support
- Language/cultural differences
- Mistrust in the healthcare system
- Poor health literacy and education

*(Probes: Can you describe a time when [insert barrier] created a particularly difficult situation? How did this impact…? What do you mean by...? Do you see different barriers at different institutions?)*

**Section 2. Understand the provider’s role and the coordination required to mitigate care disparities.**

Now, we’d like to learn more about how you and your team collaborate to help patients with GI cancer and how this work affects you and your colleagues.

Who would you say is part of your team?

*(Probes: Who do you interact with most closely when coordinating care? Are there others involved less directly?)*

Can you describe a time when **coordinating care for a patient as a team was particularly difficult**? What factors contributed to the challenges in that situation?

*(Probes: What do you mean by...? Can you tell me why…? Can you give me an example of…?)*

Can you share an example of a time **when your team working together made a real difference for a patient**?

*(Probes: What worked well? What helped the team coordinate care effectively?)*

When you’re helping patients navigate these barriers, what feels **most frustrating**? What do you wish **worked better**?

*(Probes: What could be improved with better systems, workflows, or support?)*

Of all the challenges you’ve mentioned, are there any that feel particularly **urgent** or **high-impact** to address? That, if fixed, would make a meaningful difference for patients or your team?

*(Probes: Why that one? What kind of change do you think would make the biggest impact?)*

Do you feel you were adequately trained or prepared to help patients navigate these kinds of non-medical challenges?

*(Probes: What training or support have you received, if any? What would have helped you feel more prepared?)*

How do these responsibilities affect your **job satisfaction** or **overall well-being**?

*(Probes: Do you feel like this contributes to burnout? Have you seen this impact your colleagues? Can you give me an example of…?)*

**Section 3. Identify actionable strategies and resources to address non-medical barriers to care.**

We want to **develop a multidisciplinary program to support patients undergoing cancer treatment**. Think of it as a “tumor board” focused on non-medical patient issues rather than cancer and clinical decision-making. This would bring together social workers, patient navigators, and other team members to work alongside the oncology team.

How might this help your patients?

How might it support you and your team?

*(Probes: Could something like this reduce burnout or make your work feel more sustainable?)*

If you were designing this program, what would be the most important elements?

*(Probes: What do you mean by...? Can you tell me how…? Why do you believe this…?)*

**Conclusion**

Before we wrap up, is there anything else from your experience that you think would help us better understand the barriers and facilitators of GI cancer care?

We know that providers like you work hard and are often under a lot of stress. It can feel like so much is being asked of you. Thank you so much for your time and willingness to share today. We truly appreciate your perspective and participation. Please feel free to reach out if you have questions, comments, or concerns.

[end of interview]
